# Cognitive training, exercise training or combined training? A comparative effectiveness research study on subjective and objective cognitive outcomes in multiple sclerosis

**DOI:** 10.1007/s00415-025-13535-w

**Published:** 2026-01-16

**Authors:** Naomi Gyger, Tobias Monschein, Melanie Filser, Sharon Bätge, Alina Renner, Melanie Roth, Orhan Aktas, Philipp Albrecht, Hans-Peter Hartung, Iris-Katharina Penner

**Affiliations:** 1 Department of Neurology, Inselspital, Bern University Hospital, University of Bern, Freiburgstrasse 37, 3010 Bern, Switzerland; 2 Department of Neurology, Medical University of Vienna, Vienna, Austria; 3 Comprehensive Center for Clinical Neurosciences and Mental Health, Medical University of Vienna, Vienna, Austria; 4 COGITO Center for Applied Neurocognition and Neuropsychological Research, Düsseldorf, Germany; 5 Department of Neurology, Medical Faculty, University Clinic and Heinrich-Heine-University of Düsseldorf, Düsseldorf, Germany; 6 Department of Neurology, Maria Hilf Clinics, Mönchengladbach, Germany; 7 Brain and Mind Centre, University of Sydney, Sydney NSW, Australia; 8 Department of Neurology, Palacky University Olomouc, Olomouc, Czechia; 9 Department of Experimental Psychology, Heinrich Heine University, Düsseldorf, Germany

**Keywords:** Multiple sclerosis, Cognitive impairment, Cognitive rehabilitation, Exercise training, Neurorehabilitation

## Abstract

**Objective:**

Cognitive impairment (CI) in people with multiple sclerosis (pwMS) may be present from the onset, is common, and has a profound impact on those affected. This study compared the effectiveness of three different therapeutic approaches (computerized cognitive training, BrainStim (BS); treadmill walking (TW); combined training (BS + TW)) on subjective and objective cognitive functioning in pwMS and CI.

**Methods:**

61 pwMS were recruited from the Department of Neurology outpatient clinic of the University Hospital Düsseldorf and neurologists in private practice. After screening for eligibility, pwMS were randomized into three treatment arms: 1) BS, 2) TW, 3) BS + TW. Subjective and objective cognitive functioning were evaluated before and after intervention, including the Perceived Deficits Questionnaire (PDQ-20) and Symbol Digit Modalities Test (SDMT) as primary end points, respectively.

**Results:**

46 pwMS entered the final analysis (n = 15 excluded). Change scores revealed significant effects on PDQ20 total score for all three treatment groups. These effects were stable over 6 months in the BS + TW group only. Moreover, change scores revealed significant effects on the SDMT for the TW group and BS + TW group, lasting 6 months. Between-group differences were not significant.

**Conclusion:**

Comparing three different behavioral treatments, we found that exercise training (TW), computer-based cognitive training (BS), and their combination (BS + TW) significantly improved subjective cognitive performance, with the combined group showing the most long-lasting effect. Objective cognitive performance improved significantly in the TW and BS + TW group, while cognitive training alone showed no effect. Results suggest that combining exercise with cognitive training may provide additional cognitive benefits than either intervention alone.

**Supplementary Information:**

The online version contains supplementary material available at 10.1007/s00415-025-13535-w.

## Introduction

Multiple sclerosis (MS) is a chronic inflammatory disease of the central nervous system, characterized by distinct clinical subtypes and a wide range of symptoms. Hidden symptoms of MS—such as cognitive impairment (CI), fatigue, depression, and anxiety—are often overlooked, despite their profound impact on social life, work ability, and overall quality of life [1, 2]. CI affects approximately 50% of people with MS (pwMS), often from the earliest stages of the disease [3]. Cognitive function is especially crucial for maintaining employment, which has significant implications both individually and economically [2, 4, 5]. Early cognitive screening, beginning at diagnosis, along with regular follow-up (e.g., annually), is therefore essential [6, 7]. Patient-reported outcomes (PROs) have recently become more prominent, supporting both personalized care and cost-effective treatment decisions [8, 9]. An established PRO for the subjective assessment of cognitive performance in pwMS is the Perceived Deficits Questionnaire (PDQ-20) [10]. Decreased Information processing speed (IPS) is often the earliest and most frequently reported cognitive deficit in pwMS. IPS is closely linked to broader cognitive performance and is predictive of various other outcomes (e.g., vocational status, driving ability, instrumental activities of daily living, overall quality of life) [11–13]. Although reliable diagnostic tools exist to assess IPS and overall cognitive status in MS, there remains an unmet need for effective treatment strategies. A recent review found that there is insufficient evidence supporting the efficacy of pharmacological treatments for improving cognitive function in pwMS [14]. In contrast, a review of non-pharmacological approaches indicates that exercise training can lead to significant improvements in cognitive function in pwMS [15]. Exercise training in people with MS is recognized as a potential disease-modifying intervention [16, 17]. While evidence for improvements in walking outcomes following exercise is robust, further research is needed to confirm benefits on cognitive functioning [18, 19]. So far, a number of studies have shown improvements in processing speed, learning and memory performance, as well as executive functions, after exercise training [20, 21]. One study specifically compared the acute effects of different exercise modalities (moderate-intensity treadmill walking, moderate-intensity cycle ergometry and guided yoga) and concluded that pwMS had the largest beneficial effects from treadmill walking [22]. Beyond clinical findings, animal studies also provide support for biological plausibility, showing that aerobic exercise enhances neuroplasticity and may therefore offer therapeutic benefits for pwMS [23, 24]. In addition to exercise, cognitive rehabilitation is currently considered one of the most promising approaches for improving and maintaining both motor and cognitive functions in pwMS [25]. A recent study by Feinstein et al. investigated aerobic exercise and cognitive rehabilitation in people with progressive MS (pwPPMS)[26]. Although all groups showed improvements in processing speed, the combined intervention did not provide additional benefits. This may be due to the more advanced neurodegeneration in pwPPMS, including greater axonal loss and cortical atrophy, which can limit the brain’s capacity to benefit from the synergistic effects of combined therapies. In contrast, people with relapsing–remitting MS (pwRRMS) generally have higher neurological reserve and more intact compensatory mechanisms, making them more likely to experience additive or synergistic cognitive and motor improvements from combined interventions [25, 27, 28]. Based on this rationale, our study focused on pwRRMS to compare the effects of computerized cognitive training, treadmill walking, and their combination on the primary outcome measures IPS and self-reported cognitive deficits. We hypothesized that the combined intervention would yield superior improvements in these measures compared to either training alone after the 12-week intervention. Additionally, the sustainability of these benefits was evaluated through a 6-month follow-up assessment.

## Methods

### Participants

61 people with relapsing–remitting MS (pwRRMS) as defined by the revised 2017 McDonald criteria [29] or people with secondary progressive MS (pwSPMS) as defined by the criteria of Lorscheider and colleagues 2016 [30] were consecutively enrolled in the study from October 2016 to September 2018. Participants were recruited through the distribution of study information and direct referrals from healthcare professionals. Enrollment took place at the outpatient clinic of the Department of Neurology at the University Hospital Düsseldorf and at large neurological practices in the North Western Rhine region. Eligible participants were aged 18–60 years, had an Expanded Disability Status Scale (EDSS) score ≤ 6.0, and a Symbol Digit Modalities Test (SDMT) z-score between − 0.2 and − 3.0. An SDMT threshold of − 0.5 to − 3.0 was originally planned, but this proved unfeasible in the ambulatory setting due to recruitment constraints. A more lenient cutoff of − 0.2 was therefore applied to include individuals at risk for cognitive impairment, consistent with evidence that patients above the conventional impairment threshold may still present with subtle cognitive deficits [31]. SDMT z-scores were calculated using the normative reference values established by Scherer et al., with adjustments for age and education as specified in their regression-based normative equations [32]. Exclusion criteria included the presence of an acute neurological or psychiatric disorder (other than MS), a recent relapse, changes in immunomodulatory treatment within the past month, or recent steroid therapy. Due to the treadmill walking intervention, individuals with cardiovascular disease were also excluded.

### Approval, registrations, and patient consents

This study was approved by the Ethics Committee of the Heinrich Heine University Düsseldorf (Study Nr: 5531R, registration-ID: 2016055083). We affirm that all methods described in this study were carried out in strict accordance to the relevant guidelines and regulations and the Declaration of Helsinki. The study was not preregistered.

### Study design

This study was designed as a randomized, superiority trial with blinding of the statistician. Patients who met the eligibility criteria, expressed interest in participation, and provided written informed consent were invited to undergo neuropsychological screening. All neuropsychological assessments were conducted at the COGITO Center Düsseldorf. After the neuropsychological baseline (BL) assessment consisting of a semi-structured interview, various neuropsychological tests, and patient-reported outcomes (PROs) (Table 1), patients were randomly assigned to one of the three intervention groups (I. working memory training with BrainStim (BS), II.) treadmill walking (TW), combination training (BS + TW)). Following the 12-week intervention phase, all participants underwent a retest (RT) using parallel versions of the neuropsychological tests applied at BL where available, as well as the same patient-reported outcomes (PROs). A follow-up (FU) assessment was conducted 6 months after the intervention to evaluate the sustainability of any observed improvements. To capture potential behavioral changes following the intervention, participants were asked at each follow-up whether their level of physical and cognitive training activity had changed since the intervention period. Specifically, they were asked whether they engaged in more or less physical or cognitive training compared to during the program, and, if applicable, to indicate the approximate number of hours per week devoted to these activities. These self-reported data allowed us to monitor changes in lifestyle factors that could potentially influence cognitive or functional outcomes after the training period. Data were pseudonymously encoded using numerical codes and subsequently entered into an electronic database. To ensure quality, data was cross-checked after the initial entry.

**Table 1:** O﻿utcome measures including self-report questionnaires and neuropsychological tests

End point	Outcome measure	Domain/description
Primary		
*Subjective*	PDQ-20 [33]	Perceived cognitive deficits across 4 domains: attention/concentration, retrospective memory, prospective memory, and organization/planning
*Objective*	SDMT	Information processing speed (IPS); part of BICAMS [34, 35]
Secondary		
*Subjective*	FSMC [36]	Fatigue (motor and cognitive)
	HADS [37]	Anxiety and depression
	PSS [38]	Perceived stress
	CSES [39]	Coping behavior/self efficacy
*Objective*	Other parts of BICAMS including: [34, 35]	
	VLMT	Verbal learning and short-term memory
	BVMT-R [34, 35]	Visuospatial learning and short-term memory
	TMT including: [40] TMT-A [40] TMT-B [40]	Speed (TMT-A), executive function and flexibility (TMT-B)
	Digit Span Forward and Backward (Wechsler-Memory Scale) [41]	Verbal short-term memory (forward) and working memory (backward)
	Corsi Block Span Forward and Backward (Wechsler-Memory Scale) [41]	Visuospatial short-term memory (forward) and working memory (backward)
	RWT [42]	Executive function: verbal fluency
	D-KEFS Tower Test [43]	Executive function: planning and problem-solving abilities
	MWT) [44]	Vocabulary-based intelligence

*BICAMS* Brief International Cognitive Assessment for MS; *BVMT-R* Brief Visuospatial Memory Test-Revised; *CSES* Coping Self-Efficacy Scale; *FSMC* Fatigue Scale for Motor and Cognitive Functions; *HADS* Hospital Anxiety and Depression Scale; *PDQ-20* Perceived Deficit Questionnaire; *PSS* Perceived Stress Scale; *SDMT* Symbol Digit Modalities Test; *VLMT* Verbal Learning and Memory Test; *RWT* Regensburger Verbal Fluency Test; *TMT* Trail Making Test; *D-KEFS* Delis–Kaplan Executive Function System; *MWT* Mehrfach-Wortschatz-Test. All instruments were administered at baseline, post-intervention (retest), and 6-month follow-up. The MWT was conducted only at baseline. Alternative test versions were used when available to reduce learning effects

### Outcome measures

The study assessed both subjective and objective primary and secondary outcomes (see Table 1 for an overview). The two primary end points captured subjective and objective dimensions of cognitive functioning. The primary subjective outcome was perceived cognitive deficits, assessed with the PDQ-20 [33]. The primary objective outcome was IPS, measured with the SDMT, which is part of the Brief International Cognitive Assesment for Mutiple Sclerosis (BICAMS) battery [45]. The SDMT provides an objective measure of IPS and is sensitive to disease activity, progression, and treatment effects [46, 47]. It's important to note thatsubjective (e.g PROs) and objective assessments of cognition often diverge. Specifically, subjective reports are influenced not only by actual cognitive impairment, but also by factors such as depression and fatigue.

Secondary outcomes covered multiple domains. Subjective outcomes included fatigue, assessed with the Fatigue Scale for Motor and Cognitive Functions (FSMC [36]); mood, assessed with the Hospital Anxiety and Depression Scale (HADS [37]); perceived stress, assessed with the Perceived Stress Scale (PSS [38]); and coping/self-efficacy, assessed with the Coping Self-Efficacy Scale (CSES [39]). Objective secondary outcomes included other BICAMS subtests (California Verbal Learning Test-II/RAVLT for verbal learning and memory, and the Brief Visuospatial Memory Test-Revised [BVMT-R [40]] for visuospatial memory). Additional cognitive domains were assessed using the Trail Making Test A and B (TMT-A/B [40]), Digit Span Forward/Backward and Corsi Block Span Forward/Backward [41], Regensburger Word Fluency Test (RWT [42]), and D-KEFS Tower Test [43]. Vocabulary-based premorbid intelligence was assessed with the Mehrfachwahl-Wortschatz-Test (MWT [44]).

### Interventions

#### BrainStim: cognitive training

Participants in the BS group received BrainStim, a computerized, standardized, and manualized working memory training program based on Baddeley’s working memory model [48]. BrainStim was developed by the senior author [49] and is one of the few existing cognitive training tools whose efficacy has been evaluated across a range of brain disorders—including juvenile and adult-onset MS, Parkinson’s disease, and schizophrenia—as well as in healthy controls [50–54]. The program’s three modules—City Map, Find Pairs, and Memorise Numbers—have been shown to train various aspects of working memory, leading to positive effects on working memory and IPS in pwMS [53]. Although the SDMT primarily measures IPS, its performance relies in part on working memory processes, such as encoding, storing, and retrieval of symbol–digit associations [55]. Therefore, a working memory-focused training was considered appropriate to target the core construct of IPS as well as related cognitive processes known to influence SDMT performance. This rationale is consistent with prior findings suggesting that multi-domain training approaches, which combine working memory and processing speed components, can lead to broader and longer-lasting cognitive benefits in people with MS [55, 56]. The City Map module focuses on verbal and visuospatial working memory, the Find Pairs module trains visual short-term and working memory as well as the updating function of the central executive, and the Memorize numbers module primarily targets linguistic aspects of working memory. Each module not only involves exercises and repetitions, but also actively engages participants in developing and consolidating effective strategies. The program is adaptive, adjusting to individual performance: after successfully completing several tasks, the difficulty level increases, whereas it decreases if participants fail to complete a set number of tasks. This adaptive approach aims to enhance both the program’s effectiveness and user acceptance.

Participants in the BS group completed two 45-min training sessions per week over a 12-week intervention period. After an introduction by a neuropsychologist, they received a USB stick with the program, allowing home-based training; for participants without an appropriate device, a study laptop was provided for the duration of the intervention. Key session parameters—such as timing, accuracy, reaction times, and difficulty—were recorded by the participants in a standardized training log to monitor adherence and performance. An example of the cognitive training log used during the intervention is included in the Supplmentary Materials (Supplementary File 3).

#### Treadmill walking: exercise training

Participants in the TW group received a complimentary gym membership at a fitness center near their residence, with initial heart rate assessed at the start of the intervention. They engaged in treadmill-based cardio training within 30–70% of their heart rate reserve (HRR), tailored to individual comfort levels and supervised by a certified fitness trainer. This range is recommended in the literature as safe and effective for people with relapsing–remitting MS and is consistent with exercise guidelines for clinical populations [57]. It also covers the levels shown in Sandroff and colleagues’ studies to improve cognitive processing speed and executive function in people with MS [22, 58]. Individual HRR was determined at baseline, and training intensity was adjusted according to tolerance and progression.

The intervention consisted of two 45-min training sessions per week over a 12-week period. Key session parameters (duration, average heart rate, and perceived exertion) were documented in standardized logs to monitor adherence and performance. Participants in the treadmill group were instructed to limit their physical activity to the prescribed treadmill sessions during the 12-week intervention period. To identify potential additional exercise, participants answered a brief questionnaire at the end of the intervention regarding any physical activity beyond the study protocol.

#### Combined approach: cognitive and exercise training in pwMS

Participants in the BS + TW group underwent a dual training approach over the 12-week intervention period, which involved cognitive training sessions twice weekly alongside exercise training sessions also held twice weekly. Each session lasted 45 min, resulting in a total of four training units per week. Participants assigned to this group completed both the cognitive training log and the treadmill training log, as they engaged in both training modalities.

The three different interventions served as mutual controls for each other within a superiority design framework, ensuring that each participant received a treatment. Prior to the intervention, a questionnaire was administered to assess participants’ weekly exercise hours, perceived physical fitness, previous treadmill experience (i.e., no experience, very little experience, fairly little experience, some experience, or a lot of experience), as well as any prior experience with cognitive training.

### Analysis

#### Sample size and power calculation

An a priori power analysis was conducted using G*Power (version 3.1.9.2) to determine the required sample size. Assuming a significance level of α = 0.05, a test power of 1 − β = 0.95, a medium effect size (f = 0.25), and an estimated correlation among the three repeated measurements of r = 0.40, the analysis indicated a required total sample size of N = 63. This target was nearly achieved in the intention-to-treat sample (N = 61), but not in the per-protocol sample (N = 46).

#### Statistical analysis

Statistical analyses were performed using RStudio software (RStudio Version 1.2.5033). Statistical significance was set at a p-value of 0.05 and reported down to 0.001. In this study, the per-protocol sample was analyzed. Descriptive results are presented according to the nature of the data as mean with standard deviation (SD), median with range, and percentages, respectively. To analyze group differences in continuous variables, ANOVAs and Kruskal–Wallis tests were performed, depending on the distribution of the data (normality assessed via the Shapiro–Wilk test). For categorical variables, Pearson’s Chi-square test was conducted.

Change scores were computed by subtracting retest (RT) from baseline (BL) mean scores per measure to examine within-group effects from onset to the end of the 12-week intervention. Within-group analyses were performed using one-sample t-tests on the change scores for each measure; when the assumption of normality was violated, Wilcoxon signed-rank tests were used.

Between-group effects were analyzed using mixed ANCOVAs with time (BL, RT, FU) as a within-subject factor and group (BS, TW, BS + TW) as a between-subject factor. Education was included as a covariate, as it differed significantly between groups. Other potential covariates (e.g., age, sex, disease duration, EDSS, employment status) did not differ significantly and were therefore not included in the models.

To address potential learning effects in repeated cognitive assessments, the main effect of time in these repeated-measures analyses captured general practice-related gains across measurement points, while time × group interactions reflected intervention-specific changes beyond such influences. To assess long-term effects of the interventions, separate paired t-tests and mixed ANCOVAs across three time points (baseline, retest, 6-month follow-up), with education as a covariate, were performed. Multiple comparisons were controlled using Bonferroni-corrected p-values (p′).

Additionally, effect sizes were calculated. Specifically, Cohen's d was computed for comparisons involving means, while the Wilcoxon effect size (denoted as r) was used for non-parametric tests such as the Wilcoxon signed-rank test. Partial η^2^ values from mixed ANCOVAs were also converted to Cohen’s d effect sizes. For Cohen’s d, values between 0.20 and 0.49 are considered small, 0.50 to 0.79 moderate, and ≥ 0.80 large. For Wilcoxon’s r, values between 0.10 and 0.30 are considered small, 0.30 to 0.50 moderate, and ≥ 0.50 large.

Partial correlations were performed to examine the specific impact of the intervention on self-perceived deficits and IPS, while controlling for potential confounders (e.g., changes in depressive and/or anxiety symptoms).

Finally, to assess clinically meaningful change in SDMT scores, two thresholds from the literature were applied. A change of ≥ 4 points has been proposed as meaningful at a group level, as it has been shown to correspond with functional outcomes such as employment status and daily functioning [46]. A more conservative threshold of ≥ 8 points is recommended, based on reliable change methodology that distinguishes true cognitive change from practice effects or measurement error [59], thereby representing clinically meaningful change at the individual level. These two cut-offs allow for interpretation of both group-based intervention effects and clinically relevant individual improvements [46, 59]. In addition to reporting the proportion of participants meeting these criteria, we conducted a post hoc exploratory responder analysis focusing on those who achieved ≥ 8-point improvements (“best responders”), examining their baseline characteristics in comparison to non-responders. This approach allowed us to explore potential factors associated with larger individual gains, although the analysis is post hoc and should be interpreted cautiously given the modest sample size.

### Data availability

Data supporting the findings of this study are available from the corresponding author upon reasonable request by a qualified researcher and upon approval by the respective ethics committee.

## Results

### Descriptive analyses

#### Sample characteristics

**Fig. 1:**
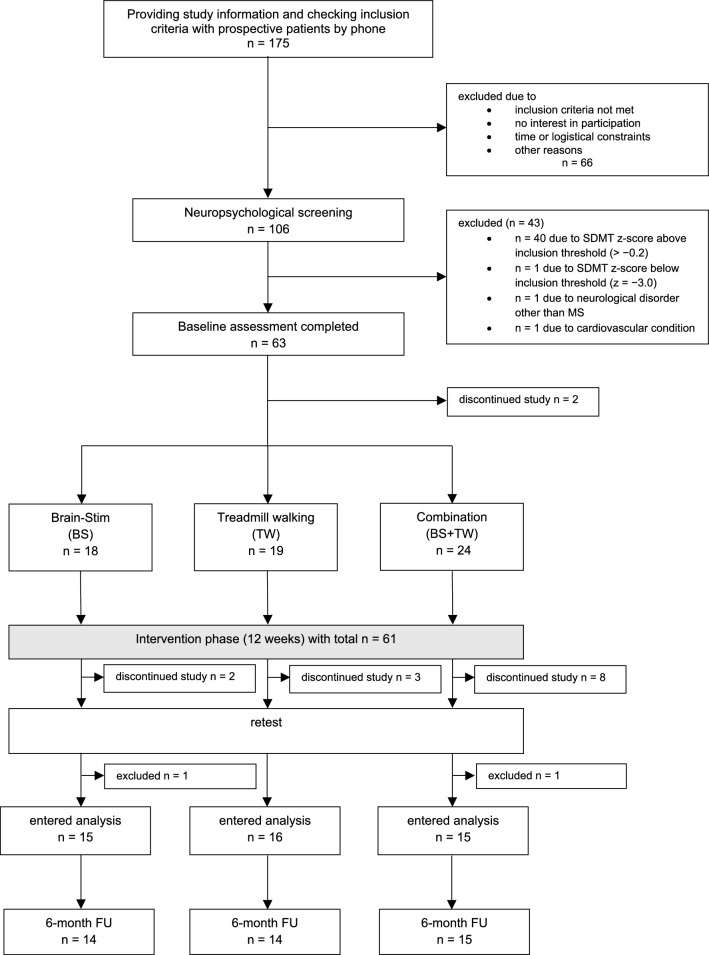
Study flowchart depicting the study's procedure: Recruitment, Screening, Baseline assessment, Treatment allocation, Retest assessment, and Follow-up assessment. It outlines exclusions and instances of participant dropouts throughout the study. In total, 13 patients discontinued the study between Baseline and Retest due to personal and health-related reasons (n = 13). Among the 48 patients who underwent Retest assessments, two patients were excluded because they completed less than 70% of the total training (n = 2). After the intervention phase of 12 weeks, Retest assessments were performed. After 6 months, Follow-Up data was collected. Not all patients attended the Follow-Up measurements, resulting in additional loss of data from three patients (n = 3). n = number of participants; FU = Follow-Up

A total of 61 participants were included in the study, representing 35% of the 175 patients who received study information. Of the 61 randomized participants, 46 (75.4%) completed more than 70% of the prescribed training sessions and were therefore included in the per-protocol analysis. Specifically, adherence rates above 70% were achieved by 16 participants in TW group, 15 in the BS group and 15 in the combined group. Two additional participants completed the intervention but did not reach the 70% adherence threshold, and the remaining dropouts were due to personal, medical, or logistical reasons. For a detailed overview of the overall study flow, including reasons for patient exclusion or discontinuation, see Fig. 1.

Table 2 presents the demographic and disease-related characteristics of the per-protocol sample (n = 46) included in the final analyses, as well as descriptive information on performance in patient-reported outcomes (PROs) and cognitive tests at baseline. There were no significant differences between the three intervention groups regarding age, sex, EDSS, disease course, disease duration, immunotherapy, employment status, or premorbid IQ. However, a significant difference in education was observed (χ^2^(4) = 12.067, p < 0.05). Moreover, no significant differences were found between intervention groups regarding scores on the applied self-report questionnaires and neuropsychological tests at baseline. Table 3 presents participants' average physical activity levels, self-reported fitness levels and previous experience with treadmill walking and cognitive training.

**Table 2:** Information on demographic and disease-related characteristics, and neuropsychological test scores at baseline

Groups	Total (N = 46)	BS (n = 15)	TW (n = 16)	BS + TW (n = 15)	p	p′
*Demographic characteristics*						
Age (y)	46.89 (8.78)	47.60 (8.81)	45.56 (8.88)	47.60 (9.10)	0.763	1.000
Sex (n; % females)	37 (80%)	11 (73%)	14 (88%)	12 (80%)	0.610	1.000
EDSS	2.85 (0.99)	2.96 (1.30)	2.63 (1.10)	2.56 (1.19)	0.616	1.000
Disease course (RRMS, n; %)	39 (85%)	12 (80%)	15 (94%)	12 (80%)	0.466	1.000
Disease duration (y)	10.20 (7.52)	10.87 (7.98)	9.47 (6.92)	10.29 (8.09)	0.877	1.000
DMT (n; % yes)	40 (87%)	14 (93%)	13 (81%)	13 (87%)	0.177	1.000
Education (n;%)						
High	32 (70%)	14 (93%)	11 (69%)	7 (47%)	0.019*	0.266
Middle	10 (21%)	1 (7%)	2 (13%)	7 (47%)	0.035*	0.490
Low	4 (9%)	0 (0%)	3 (19%)	1 (7%)	0.303	1.000
Employment (n; % yes)	24 (52%)	9 (60%)	10 (63%)	5 (33%)	0.203	1.000
Premorbid IQ (MWT)	117.52 (15.39)	121.93 (15.82)	114.25 (13.65)	116.60 (16.66)	0.374	1.000
Medications (n, %)						
Symptomatic therapies	15 (33%)	7 (47%)	5 (31%)	3 (20%)	0.398	1.000
Anxiolytics and hypnotics	1 (2%)	1 (7%)	0 (0%)	0 (0%)	1.000	1.000
Antidepressants and mood stabilizers	12 (26%)	4 (27%)	2 (13%)	6 (40%)	0.242	1.000
Analgesics	3 (6.5%)	0 (0%)	2 (13%)	1 (7%)	0.174	1.000
*Self-report questionnaire scores (mean, SD / median, IQR)*						
PDQ20 sum	31.07 (13.05)	27.13 (12.67)	33.75 (10.06)	32.13 (15.89)	0.351	n.a
FSMC total	72.85 (13.70)	70.20 (14.14)	75.31 (10.58)	72.87 (16.39)	0.594	n.a
FSMC motor	36.17 (7.06)	36.07 (7.09)	37.19 (6.06)	35.20 (8.27)	0.743	n.a
FSMC cognitive	36.67 (7.52)	34.13 (7.59)	38.13 (5.26)	37.67 (9.16)	0.282	n.a
HADS anxiety	7.67 (3.94)	6.80 (3.65)	8.44 (3.39)	7.33 (4.79)	0.522	n.a
HADS depression	5.00 (0–15)	7.00 (1–13)	9.00 (2–14)	7.00 (2–16)	0.463	n.a
CSES sum	76.04 (27.12)	80.67 (27.52)	75.56 (19.74)	74.07 (31.60)	0.776	n.a
PSS sum	24.65 (7.18)	24.33 (6.90)	25.25 (5.36)	24.33 (9.32)	0.922	n.a
*Neuropsychological test scores (mean, SD/median, IQR)*						
SDMT raw	42.91 (8.21)	44.73 (8.31)	43.69 (8.29)	40.27 (7.86)	0.301	1.000
VLMT learning	54.50 (13–73)	53.0 (45–73)	59.0 (23–71)	54.0 (13–71)	0.213	1.000
VLMT delayed recall	11.00 (2–15)	10.0 (8–15)	12.5 (2–15)	10.0 (3–15)	0.200	1.000
VLMT recognition	14 (8–15)	14.0 (9–15)	14.0 (8–15)	13.0 (8–15)	0.670	1.000
BVMT-R learning	24 (0–33)	25.0 (11–31)	21.5 (8–33)	25.0 (0–31)	0.747	1.000
BVMT-R delayed recall	10 (0–12)	11.0 (4–12)	9.5 (2–12)	10.0 (0–12)	0.748	1.000
BVMT-R recognition	6.0 (-1–6)	6.0 (5–6)	6.0 (4–6)	6.0 (-1–6)	0.057	0.912
Digit Span Forward	7.5 (3–11)	8.0 (6–11)	7.0 (4–9)	7.0 (3–11)	0.215	1.000
Digit Span Backward	6.0 (2–11)	7.0 (5–11)	6.0 (4–9)	7.0 (2–9)	0.367	1.000
Corsi Block Forward	9 (6–12)	8.0 (7–11)	9.0 (6–11)	9.0 (6–12)	0.993	1.000
Corsi Block Backward	8 (4–11)	8.0 (5–11)	7.5 (4–10)	8.0 (6–11)	0.464	1.000
TMT-A (s)	41 (18–91)	42 (19—82)	39 (18—91)	38 (25—67)	0.668	1.000
TMT-B (s)	82 (34–257)	93 (60—135)	84 (33—257)	75 (40—111)	0.227	1.000
RWT- semantic	34 (12–49)	34 (12–42)	38 (14–49)	31 (12–39)	0.130	1.000
RWT- phonematic	20.15 (7.98)	20.53 (9.27)	21.00 (8.07)	18.87 (6.88)	0.748	1.000
RWT- change	21.41 (4.74)	20.13 (4.12)	22.06 (6.24)	22.00 (3.72)	0.453	1.000

Group differences were assessed using one-way ANOVA, Kruskal–Wallis, or Chi-square tests, as appropriate. Continuous variables are presented as mean (SD) or mean (SD; range) depending on distribution; non-normally distributed variables as median (IQR); categorical variables as number–percentage. p′ denotes the p value corrected for multiple comparisons (Bonferroni–Holm). *p* < 0.05 (uncorrected). n.a. = not applicable. Abbreviations: *BVMT-R*, Brief Visuospatial Memory Test – Revised (score range 0–36; delayed recall 0–12; recognition 0–6); *CSES*, Coping Self-Efficacy Scale (score range 0–130); *D-KEFS*, Delis–Kaplan Executive Function System; *DMT*, disease-modifying therapy; *EDSS*, Expanded Disability Status Scale; *FSMC*, Fatigue Scale for Motor and Cognitive Functions (score range 0–50); *HADS*, Hospital Anxiety and Depression Scale (score range 0–21); *MWT*, Mehrfach-Wortschatz Test (used to estimate premorbid IQ); *PDQ-20*, Perceived Deficits Questionnaire (score range 0–80); *PSS*, Perceived Stress Scale (score range 0–56); *RRMS*, relapsing–remitting multiple sclerosis; *RWT*, Regensburger Verbal Fluency Test; *SDMT*, Symbol Digit Modalities Test (score range 0–110); *TMT-A*, Trail Making Test A; *TMT-B*, Trail Making Test B; BS = BrainStim, TW = treadmill walking, BS + TW = combined training

**Table 3:** Descriptive statistics for physical activity, fitness, and cognitive training prior to the intervention (N = 46)

	Groups			
	Total (N = 46)	BS (n = 15)	TW (n = 16)	BS + TW (n = 15)
*Physical Activity, Mdn (IQR)*				
Amount of sports per week (hours per week)	2.50 (3.50)	3.50 (4.50)	2.00 (3.50)	2.50 (2.10)
*Level of fitness, n (%)*				
Below average	21 (45.7%)	6 (40.0%)	8 (50.0%)	7 (46.7%)
Average	19 (41.3%)	7 (46.7%)	6 (37.5%)	6 (40.0%)
Above average	6 (13.0%)	2 (13.3%)	2 (12.5%)	2 (13.3%)
*Treadmill walking experience, n (%)*				
No experience	15 (32.6%)	5 (33.3%)	6 (37.5%)	4 (26.7%)
Almost no experience	7 (15.2%)	2 (13.3%)	2 (12.5%)	3 (20.0%)
A little experience	13 (28.3%)	2 (13.3%)	7 (43.8%)	4 (26.7%)
Experienced	7 (15.2%)	5 (33.3%)	0 (0.0%)	2 (13.3%)
Very experienced	3 (6.5%)	1 (6.7%)	1 (6.3%)	1 (6.7%)
*Cognitive training experience, n (%)*				
Yes	27 (58.7%)	11 (73.3%)	7 (43.8%)	9 (60.0%)
No	19 (41.3%)	4 (26.7%)	9 (56.3%)	6 (40.0%)
*Frequency of cognitive training, n (%)*				
Currently none	36 (78.3%)	10 (66.7%)	13 (81.3%)	13 (86.7%)
Daily	1 (2.2%)	0 (0.0%)	1 (6.3%)	0 (0.0%)
Weekly	6 (13.0%)	2 (13.3%)	2 (12.5%)	2 (13.3%)
Monthly	1 (2.2%)	1 (6.7%)	0 (0.0%)	0 (0.0%)
Yearly	2 (4.3%)	2 (13.3%)	0 (0.0%)	0 (0.0%)

Percentages are calculated based on the number of participants within each group (BS = BrainStim, TW = treadmill walking, BS + TW = combined training)

### Inferential analyses of baseline, retest, and follow-up data

Table 4 summarises the inferential analyses for changes from BL to RT and from BL to FU across all outcome measures.

**Table 4:** Change scores in test values

	Groups											
	BS (n = 15)				TW (n = 16)				BS + TW (n = 15)			
	Change score mean (SD)	p	p’	Effect size	Change score mean (SD)	p	p’	Effect Size	Change score mean (SD)	p	p’	Effect Size
*Self-report questionnaire scores*												
PDQ-20 sum												
RT-BL	− 4.80	0.018*	0.414	0.43	− 3.94	0.028*	0.476	0.39	− 5.60	0.004**	0.084	0.39
FU-BL	0.00	0.500	1.000	0.00	− 3.69	0.040*	0.600	0.32	− 7.40	0.012*	0.204	0.52
Attention/concentration												
RT-BL	− 1.20	0.075	1.000	0.37	− 1.19	0.025*	0.456	0.36	− 2.00	0.001**	0.024	0.48
FU-BL	− 0.07	0.453	1.000	0.02	− 1.00	0.064	0.640	0.28	− 2.40	0.004**	0.084	0.58
Retrospective memory												
RT-BL	− 1.40	0.013*	0.312	0.40	− 1.19	0.049*	0.611	0.42	− 2.07	0.003**	0.066	0.54
FU-BL	0.07	0.529	1.000	0.02	− 0.31	0.337	1.000	0.09	− 2.98	0.001**	0.024	0.79
Prospective memory												
RT-BL	− 1.33	0.023*	0.506	0.60	− 1.19	0.009**	0.198	0.48	− 0.93	0.042*	0.630	0.30
FU-BL	0.00	0.500	1.000	0.00	− 1.06	0.005**	0.120	0.37	− 0.67	0.194	1.000	0.22
Planning/organization												
RT-BL	− 0.87	0.184	1.000	0.26	− 0.38	0.252	1.000	0.11	− 0.60	0.179	1.000	0.13
FU-BL	0.00	0.500	1.000	0.00	− 1.31	0.047*	0.611	0.37	− 1.33	0.122	1.000	0.29
FSMC-total												
RT-BL	− 2.33	0.121	1.000	0.18	− 4.69	0.040*	0.600	0.36	− 0.73	0.357	1.000	0.05
FU-BL	2.47	0.194	1.000	0.16	− 4.44	0.005**	0.120	0.36	− 6.07	0.011*	0.198	0.38
FSMC-motor												
RT-BL	− 0.87	0.229	1.000	0.17	− 2.88	0.047*	0.611	0.39	− 0.80	0.213	1.000	0.10
FU-BL	1.27	0.150	1.000	0.22	− 2.06	0.011*	0.231	0.42	− 3.80	0.009**	0.171	0.62
FSMC-cognitive												
RT-BL	− 1.47	0.110	1.000	0.18	− 1.81	0.074	0.666	0.29	0.07	0.520	1.000	0.01
FU-BL	1.20	0.268	1.000	0.14	− 2.38	0.024*	0.456	0.36	− 2.27	0.044*	0.630	0.26
HADS anxiety												
RT-BL	− 1.00	0.066	1.000	0.27	− 0.75	0.103	0.728	0.23	− 1.27	0.036*	0.576	0.31
FU-BL	− 1.07	0.093	1.000	0.31	− 1.25	0.091	0.728	0.35	− 1.87	0.065	0.845	0.44
HADS depression												
RT-BL	0.13	0.613	1.000	0.03	− 1.63	0.014*	0.280	0.58	− 0.93	0.091	1.000	0.19
FU-BL	0.07	0.544	1.000	0.02	− 1.25	0.028*	0.476	0.42	− 1.47	0.097	1.000	0.30
CSES sum												
RT-BL	3.73	0.409	1.000	0.13	4.69	0.352	1.000	0.25	6.67	0.102	1.000	0.22
FU-BL	16.69	0.371	1.000	1.03	1.73	0.142	0.852	0.10	8.67	0.076	0.912	0.49
PSS sum												
RT-BL	− 1.60	0.259	1.000	− 0.21	− 0.38	0.823	1.000	− 0.06	0.20	0.914	1.000	0.02
FU-BL	− 2.00	0.106	1.000	0.40	− 1.19	0.180	0.900	0.24	− 3.87	0.097	1.000	0.53
*Neuropsychological test scores*												
SDMT raw												
RT-BL	2.13	0.096	0.864	0.24	4.75	0.001**	0.015	0.47	5.47	0.026*	0.364	0.71
FU-BL	3.67	0.053	0.663	0.41	6.38	0.000***	0.000	0.66	5.00	0.012*	0.180	0.62
VLMT learning												
RT-BL	4.87	0.013*	0.195	0.70	2.13	0.149	1.000	0.20	7.20	0.042*	0.504	0.51
FU-BL	3.20	0.073	0.803	0.55	2.44	0.101	1.000	0.34	5.47	0.030*	0.390	0.74
VLMT delayed recall												
RT-BL	1.53	0.019*	0.247	0.57	− 0.50	0.818	1.000	0.22	1.00	0.194	1.000	0.34
FU-BL	0.40	0.285	1.000	0.19	− 0.06	0.439	1.000	0.02	0.87	0.164	1.000	0.34
VLMT recognition												
RT-BL	0.13	0.411	1.000	0.06	− 0.69	0.829	1.000	0.22	0.07	0.500	1.000	0.02
FU-BL	− 0.33	0.242	1.000	0.21	0.44	0.150	1.000	0.30	1.00	0.133	1.000	0.55
BVMT-R learning												
RT-BL	0.40	0.390	1.000	0.07	− 0.44	0.675	1.000	0.06	0.93	0.345	1.000	0.10
FU-BL	1.27	0.127	1.000	0.37	2.44	0.016*	0.224	0.58	2.93	0.034*	0.408	0.49
BVMT-R delayed recall												
RT-BL	0.20	0.472	1.000	0.02	0.69	0.128	1.000	0.22	0.33	0.742	1.000	0.20
FU-BL	0.40	0.212	1.000	0.18	0.81	0.110	1.000	0.27	1.27	0.016*	0.224	0.32
Digit Span Forward												
RT-BL	− 0.47	0.895	1.000	0.32	0.0	0.443	1.000	0.13	1.33	0.001***	0.015	0.56
FU-BL	− 0.31	0.599	1.000	0.08	− 0.19	0.710	1.000	0.11	− 0.13	0.434	1.000	0.08
Digit Span Backward												
RT-BL	− 0.07	0.573	1.000	0.04	− 0.69	0.973	1.000	0.46	0.33	0.186	1.000	0.18
FU-BL	− 0.40	0.751	1.000	0.23	− 0.44	0.862	1.000	0.29	0.53	0.075	0.825	0.29
Corsi Block Forward												
RT-BL	0.60	0.029	0.348	0.54	0.19	0.327	1.000	0.11	0.13	0.403	1.000	0.06
FU-BL	0.53	0.020*	0.280	0.58	− 1.00	0.998	1.000	− 0.87	0.27	0.308	1.000	0.13
Corsi Block Backward												
RT-BL	0.67	0.058	0.580	0.43	0.50	0.067	0.938	0.40	0.40	0.212	1.000	0.21
FU-BL	− 0.20	0.696	1.000	− 0.14	0.25	0.261	1.000	0.16	− 0.27	0.678	1.000	− 0.12
TMT-A												
RT-BL	− 4.69	0.049*	0.539	0.36	− 3.51	0.143	1.000	0.21	− 3.98	0.102	1.000	0.39
FU-BL	− 4.69	0.017*	0.255	0.36	− 3.51	0.436	1.000	0.21	− 3.98	0.316	1.000	0.39
TMT-B												
RT-BL	− 12.18	0.016*	0.224	0.48	− 4.86	0.719	1.000	0.14	− 5.42	0.136	1.000	0.30
FU-BL	− 12.18	0.051*	0.663	0.48	− 4.86	0.041*	0.533	0.14	− 5.42	0.582	1.000	0.30
RWT												
Phonematic												
RT-BL	− 5.00	0.994	1.000	− 0.75	− 4.69	1.000	1.000	− 1.29	− 3.33	0.995	1.000	− 0.77
FU-BL	− 4.38	0.986	1.000	0.63	− 4.16	0.998	1.000	− 0.88	− 3.13	0.982	1.000	− 0.60
Semantic												
RT-BL	1.60	0.254	1.000	0.18	− 0.75	0.656	1.000	− 0.10	4.27	0.029*	0.377	0.53
FU-BL	− 7.29	1.00	1.000	− 1.22	− 8.05	0.999	1.000	− 0.99	− 6.13	0.996	1.000	− 0.80
Change												
T-BL	0.20	0.463	1.000	0.02	− 1.81	0.965	1.000	− 0.49	− 3.60	0.990	1.000	− 0.67
FU-BL	0.91	0.206	1.000	0.22	− 2.24	0.963	1.000	− 0.48	− 1.00	0.774	1.000	− 0.20

Data are mean (SE). *BVMT-R* Brief Visuospatial Memory Test revised (total score range = 0–36; delayed recall score range = 0–12; recognition score range = 0–6), *CSES* Coping Self-Efficacy Scale (total score range: 0–130), Corsi Block Forward visuospatial short-term memory (total score range = 0–12), Corsi Block Backward visuospatial working memory (total score range = 0–12), Digit Span Forward: verbal short-term memory (total score range = 0–12), Digit span backward: verbal working memory (total score range = 0–12), *EDSS* Expanded Disability Status Scale, *FSMC* Fatigue Scale for Motor and Cognitive Functions (subscale score ranges = 0–50), *HADS* Hospital Anxiety and Depression Scale (subscale score ranges = 0–21), *PDQ-20* Perceived Deficit Questionnaire (total score range = 0–80), *PSS* Perceived Stress Scale (total score range = 0–56), *SDMT* Symbol Digit Modalities Test (total score range = 0–110), *VLMT* Verbal Learning and Memory Test (total score range = 0–75, delayed recall score range = 0–15, recognition score range = − 20–15), *RWT* Regensburger Verbal Fluency Test, *TMT-A* Trail Making Test A, *TMT-B* Trail Making Test B. Change scores are represented by the raw values obtained from neuropsychological tests or questionnaires. Positive change score in neuropsychological test scores and *CSES* (self-efficacy) describes an improvement measured after the interventional phase. Positive change scores in *TMT-A* and *B* and all other questionnaires indicate a deterioration in the domain of interest (e.g., increased fatigue, depression etc.). p relates to within-group comparisons (Student’s t-test or Wilcoxon signed-rank); p′ resembles the p value corrected for multiple testing using the Bonferroni–Holm method.BS = BrainStim; TW = treadmill walking; BS + TW = combined training. BL = baseline; RT = retest; FU = follow-up

#### Primary outcome measures

In the BS group, PDQ-20 scores significantly decreased at retest (t(14) = − 2.328, p = 0.018, d = 0.43), with improvements on the retrospective memory (t(14) = − 2.505, p = 0.013, d = 0.40) and prospective memory subscales (t(14) = − 2.197, p = 0.023, d = 0.60). No significant improvement was observed on the SDMT (t(14) = 1.372, p = 0.096, d = 0.23).

In the TW group, participants showed a significant reduction in perceived cognitive deficits at retest, as measured by the PDQ-20 total score (t(15) = − 2.074, p = 0.028, d = 0.39), with improvements across three subscales: attention/concentration (t(15) = − 2.132, p = 0.025, d = 0.36), retrospective memory (t(15) = − 1.767, p = 0.049, d = 0.42), and prospective memory (t(15) = − 2.643, p = 0.009, d = 0.48). The TW group also demonstrated significant gains in SDMT performance (t(15) = 3.648, p = 0.001, d = 0.47), indicating improved IPS.

Given the simultaneous reduction in depressive symptoms, partial correlation analyses were conducted to examine whether improvements in PDQ-20 and SDMT were attributable to changes in depression. Results showed negligible and nonsignificant associations (PDQ-20: r = 0.070, p = 0.742; SDMT: r = –0.036, p = 0.814), indicating that improvements in the primary outcomes were not primarily driven by changes in depressive symptoms.

In the BS + TW group, PDQ-20 total scores significantly decreased at retest (t(14) = − 3.081, p = 0.004, d = 0.39), with improvements across three subscales: attention/concentration (t(14) = − 4.183, p = 0.001, d = 0.48), retrospective memory (t(14) = − 3.212, p = 0.003, d = 0.54), and prospective memory (t(14) = − 1.859, p = 0.042, d = 0.30). Anxiety symptoms also decreased significantly (HADS-A: t(14) = − 1.946, p = 0.036, d = 0.31). Regarding objective outcomes, SDMT scores improved significantly (t(14) = 3.302, p = 0.003, d = 0.71), indicating enhanced IPS.

Across all groups, Kendall’s Tau revealed a weak, non-significant correlation between changes in SDMT and PDQ-20 scores (τ = 0.17, p > 0.5), suggesting limited correspondence between subjective and objective measures.

### Secondary outcome measures

In the TW group, fatigue significantly improved, as reflected in the FSMC total score (t(15) = − 1.878, p = 0.040, d = 0.36) and motor subscale (t(15) = − 1.783, p = 0.047, d = 0.39). Depressive symptoms also decreased significantly (HADS-D: t(15) = − 2.425, p = 0.014, d = − 0.58).

In the BS group, significant gains were observed in verbal learning (VLMT learning: t(14) = 2.472, p = 0.013, d = 0.70) and delayed recall (t(14) = 2.295, p = 0.019, d = 0.57). Improvements were also found in visuospatial short-term memory (Corsi Forward: t(14) = 2.073, p = 0.029, d = 0.45), and in processing speed and cognitive flexibility (TMT-A: t(14) = − 1.778, p = 0.049, d = 0.36; TMT-B: t(14) = − 2.389, p = 0.016, d = 0.48).

In the BS + TW group, additional significant improvements were observed in verbal learning (VLMT learning: t(14) = 1.866, p = 0.045, d = 0.51) and verbal short-term memory (Digit Span forward: t(14) = 4.183, p = 0.001, d = 0.56). 

### Inferential analyses between baseline and follow-up

Table 4 presents the analysis of both primary outcomes (PDQ-20 total score and SDMT total score) and secondary outcomes at BL, and 6-month FU. Regarding the subjective primary outcome, the PDQ-20 total score significantly decreased from BL to FU in the TW group with a small effect size (*t*(15) = -1.872, *p* = 0.040, *d* = 0.32), and in the BS + TW group with a moderate effect size (*t*(14) = -2.524, *p* = 0.012, *d* = 0.52). In contrast, the BS group showed no sustained benefit, as scores returned to baseline levels at FU (*t*(14) = 0, *p* = 0.500, *d* = 0.00).

**Fig. 2:**
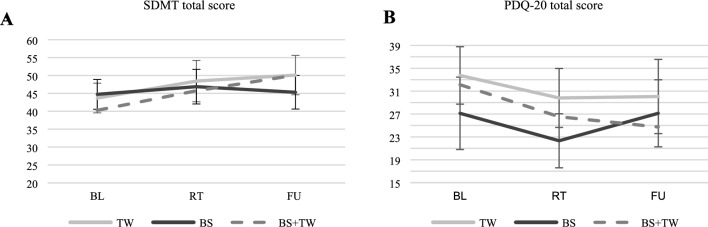
A higher score on the SDMT reflects enhanced IPS while a higher score on the PDQ-20 indicates greater perceived cognitive deficits.. Error bars represent the standard deviation. p-values are derived from one-sided tests with statistical significance defined as p < 0.05. RT = rRetest, BL = baseline, FU = follow-up, BS = BrainStim, TW = treadmill walking, BS + TW = combined training

For the objective primary outcome, SDMT scores significantly improved from BL to FU in both the TW group (*t*(15) = 5.015, *p* < 0.001, *d* = 0.82) and the BS + TW group (*t*(14) = 2.549, *p* = 0.012, *d* = 0.86). No significant SDMT change was observed in the BS group across all three time points. Among secondary outcomes, the FSMC total score significantly decreased in the TW group between RT and FU (*t*(15) = -2.937, *p* = 0.005, *d* = 0.36), and also in the BS + TW group when comparing BL to FU (*t*(15) = -2.560, *p* = 0.011, *d* = 0.38). Anxiety symptoms, as assessed by the HADS anxiety subscale, significantly decreased from BL to RT in the BS + TW group but were not sustained at FU (*t*(15) = -1.401, *p* = 0.091, *d* = -0.35). In contrast, depressive symptoms significantly decreased from BL to FU in the TW group (*t*(15) = -2.076, *p* = 0.028, *d* = -0.42). The BS + TW group also demonstrated a significant improvement in verbal learning (VLMT learning) from BL to FU (*t*(15) = 2.037, *p* = 0.030, *d* = 0.74). However, in the BS group, VLMT delayed recall scores significantly declined from RT to FU (*t*(14) = -2.241, *p* = 0.042, *d* = -0.39), and no longer showed a significant PDQ-20 benefit from BL to FU (*t*(14) = 0.581, *p* = 0.285, *d* = 0.19). Significant improvements in the BVMT-R total score were observed in both the TW group (*t*(15) = 2.370, *p* = 0.016, *d* = 0.58) and the BS + TW group (*t*(14) = 1.984, *p* = 0.034, *d* = 0.49) from BL to FU. Additionally, the BS + TW group showed a significant improvement in BVMT-R delayed recall (*t*(14) = 2.391, *p* = 0.016, *d* = 0.32). For visuospatial working memory, a significant improvement in the Corsi Block Backward task from FU to RT was found in the BS group (*t*(14) = -2.476, *p* = 0.027, *d* = 0.55), though this was not maintained when comparing BL to FU (*t*(14) = -0.526, *p* = 0.696, *d* = 0.12). In the TW group, performance on the Corsi Block Forward task significantly declined from FU to RT (*t*(15) = -2.967, *p* = 0.010, *d* = -0.79), and from BL to FU (*t*(14) = -3.464, *p* = 0.002). Processing speed and flexibility, assessed via the Trail Making Test, also showed significant improvements: TMT-A scores improved in the BS group from BL to FU (*t*(14) = -2.364, *p* = 0.017, *d* = 0.48), while TMT-B scores improved in the TW group (*t*(15) = -1.861, *p* = 0.041, *d* = 0.31). Importantly, when adjusting for education, no significant interaction between time and group was found for any outcome measure across BL and FU. Figure 2 displays the change in the two primary outcomes (SDMT and PDQ-20) across baseline, retest and follow-up for each intervention group (Fig. 2). Line graphs for additional secondary outcomes showing significant-within group changes are provided in the Supplementary Materials (Supplementary File 2)

### Comparison among three interventions regarding the effects between baseline and retest investigated by mixed ANCOVAS

After adjusting for education, no statistically significant interaction between time and group was observed for any PRO or neuropsychological test, with one exception. A significant time × group interaction was found for the Digit Span Forward task, F(2, 43) = 6.797, p = 0.003, partial η^2^ = 0.316, d = 1.36. Specifically, the BS + TW group demonstrated significantly greater improvements in verbal short-term memory performance compared to the BS group. For a detailed summary of the mixed ANCOVA results, see Supplementary Materials (Supplementarty File 1) A1.

### Clinical significance

Across all groups, 25 of 46 participants (54.3%) demonstrated an improvement of ≥ 4 points on the SDMT, while 15 participants (32.6%) achieved an improvement of ≥ 8 points. The highest proportion of “best responders” (≥ 8 points) was observed in the BS + TW group (7 of 15 participants, 46.7%), followed by the TW group (3 of 16, 19.0%) and the BS group (3 of 15, 20.0%).

To explore potential predictors of response, baseline characteristics of best responders were compared to non-responders. No significant baseline differences were found between best responders and non-responders in age, education, average sleep duration, employment status, baseline SDMT or PDQ-20 scores, fatigue, depression, or anxiety. Numerically, best responders had lower EDSS scores (median = 2.5 vs. 3.0; W = 300.5, p = 0.104) and shorter disease duration (median = 72 months, IQR = 30.5–99 vs. 116 months, IQR = 50.5–181.5; W = 298, p = 0.076), although these differences did not reach statistical significance.

## Discussion

The objective of the present study was to investigate and compare the effects of three different non-pharmacological treatment approaches on self-perceived cognitive deficits and IPS in pwMS. The treatment approaches included treadmill walking, cognitive training using the computerized working memory program BrainStim and a combined approach.

As previously mentioned, there is a lack of available treatment approaches specifically indicated for pwMS with CI. Regarding pharmacological treatments, Landmeyer and colleagues conducted a systematic review and meta-analysis of longitudinal cognitive changes in tests assessing IPS. It is important to note that most of the included studies were phase III trials that did not designate CI as a primary endpoint. Overall, the results indicated a robust, small to moderate positive effect of disease-modifying therapies (DMTs) (g = 0.27). No differences were found between low- and high-efficacy DMTs [60]. For example, one study—a randomized, double-blind, placebo-controlled trial—demonstrated that patients receiving dalfampridine twice daily for 12 weeks showed a mean increase of 9.9 points in SDMT scores, compared to an increase of 5.2 points in the placebo group. Notably, this improvement was not sustained after discontinuation of the drug [61]. With regard to non-pharmacological approaches, one study demonstrated that brain-derived neurotrophic factor (BDNF) levels, dual-task performance, and emotional health outcomes were more strongly enhanced following combined cognitive-aerobic exercise training compared to aerobic exercise alone [62]. Similarly, another study found significant improvements in cognitive efficiency and health-related quality of life following cognitive-motor training compared to computer-based cognitive training alone [63].

Findings from this randomized comparative effectiveness research trial of exercise training, cognitive training and a combined training approach indicate that all three interventions significantly reduced self-perceived cognitive deficits, as measured by the PDQ-20. A greater number of PDQ-20 subscales (3 out of 4) showed significant improvement in the TW and BS + TW groups compared to the BS group (2 out of 4). Across all three groups, the PDQ-20 subscale planning/organization was the only domain that did not exhibit a notable improvement. Consistent with this, a previous pilot study reported that cognitive rehabilitation tends to yield more pronounced effects in areas such as memory and processing speed, while improvements in working memory and executive functions are generally less robust [56]. A possible explanation is that executive functions are inherently more complex and, therefore, more difficult to target and improve through training than more straightforward cognitive processes such as working memory.

While all three interventions demonstrated the potential to improve self-perceived cognitive deficits, only the BS + TW group showed further improvement over time, as observed at the 6-month FU. In contrast, a return to BL levels was observed in the BS group, which may be partially attributed to the discontinuation of access to the BrainStim training program (USB stick) after the intervention period. This limited post-intervention accessibility to cognitive training—unlike physical exercise, which is generally easier to integrate into daily routines—may have contributed to the decline, highlighting the importance of implementing booster sessions to sustain the positive effects of cognitive training. Given that CI affects daily functioning and quality of life [6], improving self-perceived cognitive deficits and cognitive functioning in pwMS may ultimately enhance overall quality of life. The significant reduction in perceived cognitive deficits across all intervention groups underscores the importance of providing pwMS with therapeutic options to improve their cognitive perceptions, irrespective of the specific type of intervention.

The findings of this study further demonstrated that both the TW and BS + TW groups exhibited significant improvements in the objective outcome of IPS as measured by the SDMT. These improvements remained stable over time in both the TW and BS + TW groups, as indicated by FU data. In contrast, the BS group did not show a significant improvement. In summary, exercise is likely the primary driver of IPS improvements in the described scenario. These findings align with existing evidence indicating that exercise training in people with MS is commonly associated with improvements in processing speed [64]. Although the underlying mechanisms were not examined in this study, prior research has proposed that exercise may promote cognitive benefits through pathways such as enhanced neuroplasticity or reductions in fatigue [65]. Given that no biological markers were collected, these processes cannot be confirmed here and should be interpreted with caution. Nonetheless, the present findings support exercise as a practical approach to improving cognitive performance in MS. However, this does not imply that cognitive training is ineffective; programs such as BrainStim remain valuable for targeting specific working memory functions, thereby underscoring the importance of individually tailored treatment strategies.

As no significant between-group differences were observed for either primary outcome, the combined approach cannot be considered superior. Several factors may help explain this finding. First, the study included patients with minimal to moderate cognitive impairment, as the SDMT threshold was adapted to facilitate recruitment in the ambulatory setting. This is noteworthy, as previous research has demonstrated that pwMS with cognitive impairment tend to experience greater cognitive benefits following exercise training [66]. Second, the overall sample size was small, and the per-protocol sample in particular did not reach the required number determined by the a priori power analysis, reducing statistical power to detect group differences. Finally, it is conceivable that each single intervention (BS or TW) produced meaningful effects on its own, thereby limiting the potential to detect further benefits of the combined approach.

Our total sample represented a patient population with rather moderate clinical manifestations due to the inclusion criteria (EDSS ≤ 6.0; SDMT z ≥ − 3.5). In contrast, the study by Feinstein et al. [26] focused on a population with progressive MS (pwPPMS), who were on average older and more severely affected at baseline, as indicated by higher EDSS scores (Median: 6 vs. Median: 3) and lower IPS (Mean SDMT: 33.4, SD: 8.2 vs. Mean: 42.91, SD: 8.2). Their findings did not demonstrate a significant benefit of the combined intervention (cognitive rehabilitation plus exercise) over single interventions or sham conditions in improving information processing speed (IPS). A possible explanation for this is the predominance of neurodegeneration and lower cognitive reserve within their sample, which may have limited the effectiveness of these approaches. In contrast, more than half of our participants showed clinically significant improvements on the SDMT, and nearly one-third reached the more stringent 8-point threshold—particularly in the TW and BS + TW groups—suggesting that intervention may be most effective when neuroplasticity and compensatory mechanisms are more active. Supporting this notion, recent studies indicate that pwMS with cognitive deficits benefit more from cognitive health interventions if initiated within five years of the onset of cognitive symptoms [67].

Our additional analysis revealed that participants who demonstrated clinically significant improvement on the SDMT—defined as an increase of 8 points or more—differed from other participants in two key factors: shorter disease duration and lower EDSS scores. These findings align with previous research indicating that neuronal plasticity declines with age and disease duration [68, 69]. Specifically, Rademacher and colleagues found that patients with lower EDSS scores exhibited greater improvements in cognitive performance—measured by verbal and visuospatial memory—following high-intensity interval training (HIIT) compared to patients with higher EDSS scores [66]. Moreover, it has been suggested that pwMS with higher cognitive reserve experience less cognitive decline than those with lower cognitive reserve. Early intervention may help preserve cognitive function in the long term or potentially delay the onset of CI [70]. These results highlight the crucial role of early diagnosis, timely treatment initiation, and ongoing disease monitoring in maintaining neurological reserve in pwMS.

## Limitations and future directions

In light of the study’s constraints, the modest sample size limits statistical power and generalizability, and therefore the findings should be interpreted with caution. Additionally, the sample consisted mainly of pwRRMS and only very few pwSPMS. However, this distinction is important, as SPMS is typically associated with more frequent and severe cognitive deficits, including greater impairments in memory, working memory, and information processing speed [71]. Notably, one study found that acute aerobic exercise activated the kynurenine pathway in pwRRMS but not in pwSPMS, indicating that metabolic responses to exercise may differ between these subtypes [72]. Thus, in future studies a distinction should be made in order to better understand the effects of the intervention among the different disease course. In addition, the analyzed sample in its final form may not fully represent all pwRRMS or SPMS but those who were eligible and motivated to participate in an outpatient intervention program. To ensure access for a wider range of pwMS and to reduce drop outs, researcher should think of ways on how to develop future outpatient group programs in a more accessible manner. However, the higher dropout rates in the combined group can primarily be attributed to the greater time and effort demands required by this intervention compared to the single-approach groups.

A further limitation relates to the influence of immunomodulatory therapies and antidepressants. Although overall immunotherapy and antidepressant use were controlled for and did not differ significantly between groups, the potential differential cognitive effects of specific DMTs and antidepressants were not examined at the individual level. Given that DMTs typically provide modest cognitive benefits and antidepressants may improve cognition indirectly through mood stabilization and neuroprotective effects, this represents an important factor in interpreting the results [73, 74].

As poor adherence is rather common in both, home based cognitive rehabilitation [75] as well as exercise-based [76], researcher have to focus on developing solutions to increase adherence.

As a current metaanalysis by Youseff et al. [77] concluded that high-intensity interval training (HIIT) is more beneficial than moderate continuous training (MCT) in improving cognitive function, specifically in terms of memory, it would be worthwhile to more specifically investigate whether varying training intensity results in different outcomes.

Another metholodigcal limitation is the use of a slightly broader SDMT inclusion range (z-score between − 0.2 and − 3.0), which was applied due to recruitment challenges in the ambulatory setting. This approach increases sensitivity and allowed the inclusion of individuals potentially at risk for cognitive impairment, but it also reduces specificity and may have resulted in the inclusion of participants without clinically relevant deficits. Another methodological limitation concerns repeated cognitive testing. Residual learning effects cannot be entirely excluded. These metholdogical issues should be considered when interpreting the cognitive outcomes.

Furthermore, future research should focus on studying physiological mechanisms that underlie the positive effect of a combined approach by including neuroimaging measures or molecular markers. In view of the limited use of cognitive rehabilitation in clinical care and the predominant use of DMTs, a direct comparison of the effect sizes of cognitive rehabilitation programmes or exercise training versus DMTs is currently unreasonable [26].

## Conclusions

In conclusion, the findings of this study indicate that all intervention groups demonstrated significant improvements in their perceived cognitive deficits, as measured by the PDQ-20. With regard to cognitive performance, as assessed by the SDMT, significant effects were observed only in the TW and BS + TW groups. These improvements were sustained through FU, indicating the potential for lasting benefits. In contrast, the BS group did not exhibit statistically significant improvements on the SDMT. This suggests that the benefits observed in the combined group were primarily driven by treadmill walking rather than additive effects of both interventions, which prevented a superiority effect. Whilst no statistically significant differences were observed between the groups, the results numerically favoured the combined approach. Specifically, the highest proportion of participants showing clinically meaningful individual improvements on the SDMT was found in the BS + TW group, which also demonstrated the most sustained improvements on the subjective measure of perceived cognitive deficits (PDQ-20). Overall, the findings indicate a possible benefit of the combined approach for both objective and subjective cognitive outcomes, despite the absence of statistically significant group-level effects. Finally, this study also holds practical significance, as it highlights the challenges which may occur when conducting intervention studies in an ambulatory setting, which demands considerable effort and resources.

## Supplementary Information

Below is the link to the electronic supplementary material.

Supplementary file1 (DOCX 16 KB)

Supplementary file2 (DOCX 1971 KB)

Supplementary file3 (DOCX 798 KB)

Supplementary file4 (DOCX 1384 KB)
