# Supplementary material for: Cognitive training, exercise training or combined training? A comparative effectiveness research study on subjective and objective cognitive outcomes in multiple sclerosis
Source: J Neurol. 2026 Jan 16;273(2):82. doi: 10.1007/s00415-025-13535-w (PMC12811355; doi:10.1007/s00415-025-13535-w)
Supplement: Supplementary file 1 — Supplementary file1 (DOCX 16 KB) [file 415_2025_13535_MOESM1_ESM.docx]

| **PROs / Neuropsychological Tests** | **Between-group Comparison (p-value)** | **Group Comparison (Significance)** | **Partial η²** | **Cohen's d** |
| --- | --- | --- | --- | --- |
| PDQ-20 sum | 0.188 | BS/TW/BS+TW n.s. | 0.008 | 0.18 |
| Attention/Concentration | 0.577 | BS/TW/BS+TW n.s. | 0.027 | 0.33 |
| Retrospective Memory | 0.531 | BS/TW/BS+TW n.s. | 0.025 | 0.32 |
| Prospective Memory | 0.148 | BS/TW/BS+TW n.s. | 0.007 | 0.17 |
| Planning/Organization | 0.164 | BS/TW/BS+TW n.s. | 0.008 | 0.18 |
| FSMC Total | 0.864 | BS/TW/BS+TW n.s. | 0.040 | 0.41 |
| FSMC Motor | 0.856 | BS/TW/BS+TW n.s. | 0.039 | 0.41 |
| FSMC Cognition | 0.689 | BS/TW/BS+TW n.s. | 0.032 | 0.36 |
| HADS Anxiety | 0.179 | BS/TW/BS+TW n.s. | 0.008 | 0.18 |
| HADS Depression | 2.128 | BS/TW/BS+TW n.s. | 0.090 | 0.63 |
| CSES Total | 1.573 | BS/TW/BS+TW n.s. | 0.073 | 0.56 |
| PSS Total | 0.314 | BS/TW/BS+TW n.s. | 0.014 | 0.24 |
| SDMT | 1.338 | BS/TW/BS+TW n.s. | 0.059 | 0.50 |
| VLMT Learning | 0.883 | BS/TW/BS+TW n.s. | 0.041 | 0.41 |
| VLMT Delayed Recall | 1.803 | BS/TW/BS+TW n.s. | 0.084 | 0.61 |
| VLMT Recognition | 0.479 | BS/TW/BS+TW n.s. | 0.022 | 0.30 |
| BVMT-R Learning | 0.185 | BS/TW/BS+TW n.s. | 0.008 | 0.19 |
| BVMT-R Delayed Recall | 0.140 | BS/TW/BS+TW n.s. | 0.006 | 0.16 |
| Digit Span Forward | 6.797 | BS < BS+TW | 0.003** | 1.36 |
| Digit Span Backward | 2.166 | BS/TW/BS+TW n.s. | 0.101 | 0.67 |
| Corsi Block Forward | 0.359 | BS/TW/BS+TW n.s. | 0.016 | 0.26 |
| Corsi Block Backward | 0.110 | BS/TW/BS+TW n.s. | 0.005 | 0.14 |
| TMT-A | 0.041 | BS/TW/BS+TW n.s. | 0.002 | 0.09 |
| TMT-B | 0.230 | BS/TW/BS+TW n.s. | 0.011 | 0.21 |

**Table A1: Between-group analysis of the three groups on cognitive tests and PROs from RT to BL**

***Note*.** Data are presented as p-value (significance), group comparison, partial η², and Cohen's d for effect size. n.s. = not significant. RT = Retest, BL = Baseline, BS = BrainStim, TW = Treadmill Walking, BS+TW = Combined Training, BVMT-R = Brief Visuospatial Memory Test Revised, CSES = Coping Self-Efficacy Scale, FSMC = Fatigue Scale for Motor and Cognitive Functions, HADS = Hospital Anxiety and Depression Scale, PDQ-20 = Perceived Deficit Questionnaire, PSS = Perceived Stress Scale, SDMT = Symbol Digit Modalities Test, VLMT = Verbal Learning and Memory Test, RWT = Regensburger Verbal Fluency Test, TMT-A = Trail Making Test A, TMT-B = Trail Making Test B.
