# Supplementary material for: Cognitive training, exercise training or combined training? A comparative effectiveness research study on subjective and objective cognitive outcomes in multiple sclerosis
Source: J Neurol. 2026 Jan 16;273(2):82. doi: 10.1007/s00415-025-13535-w (PMC12811355; doi:10.1007/s00415-025-13535-w)
Supplement: Supplementary file 2 — Supplementary file2 (DOCX 1971 KB) [file 415_2025_13535_MOESM2_ESM.docx]

**Figure A1: Self-report questionnaires and neuropsychological tests with significant changes observed in at least one comparison between baseline and retest or baseline and follow-up**

***Note*.** Data are presented for each panel showing performance across three time points: Baseline (BL), Retest (RT), and Follow-up (FU) for the following measures: HADS Anxiety, HADS Depression, VLMT Learning, VLMT Delayed Recall, FSMC Total, Digit Span Forward, Corsi Block Forward, TMT-A, and TMT-B. Error bars represent standard deviation. P-values are based on one-sided tests with significance set at p < 0.05.

The line graphs depict selected self-report questionnaires and neuropsychological tests at all time points (BL, RT, and FU) across the BS, TW, and BS+TW groups (mean and standard errors). Lower scores on HADS depression and anxiety subscales reflect reduced depression and anxiety, while higher scores in VLMT learning and recall, Digit Span Forward, and Corsi Block tasks indicate improved verbal learning, memory, and visuospatial memory. Lower scores on TMT-A and TMT-B reflect increased information processing speed.
