# Supplementary figures and images for: Cognitive training, exercise training or combined training? A comparative effectiveness research study on subjective and objective cognitive outcomes in multiple sclerosis
Source: J Neurol. 2026 Jan 16;273(2):82. doi: 10.1007/s00415-025-13535-w (PMC12811355; doi:10.1007/s00415-025-13535-w)

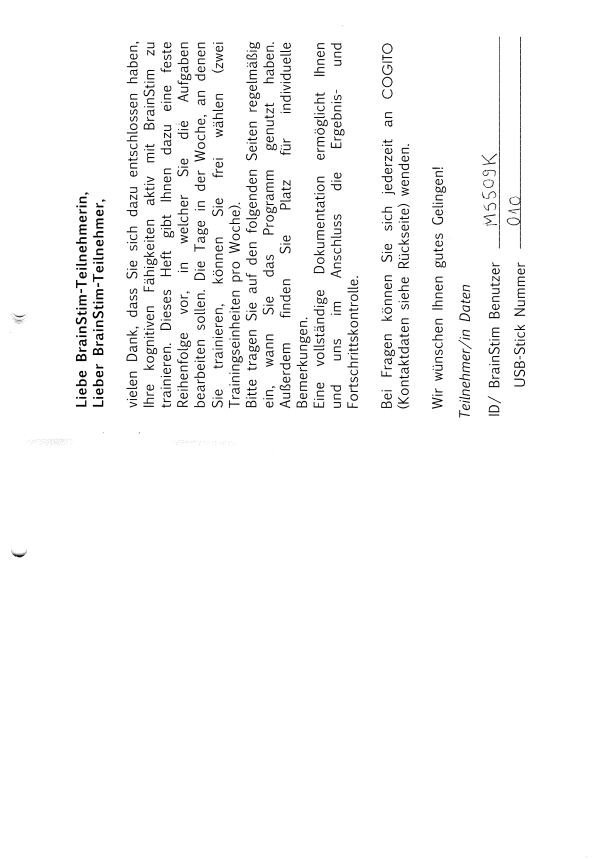
Supplementary File S1. Cognitive Training Log (Example Entry)


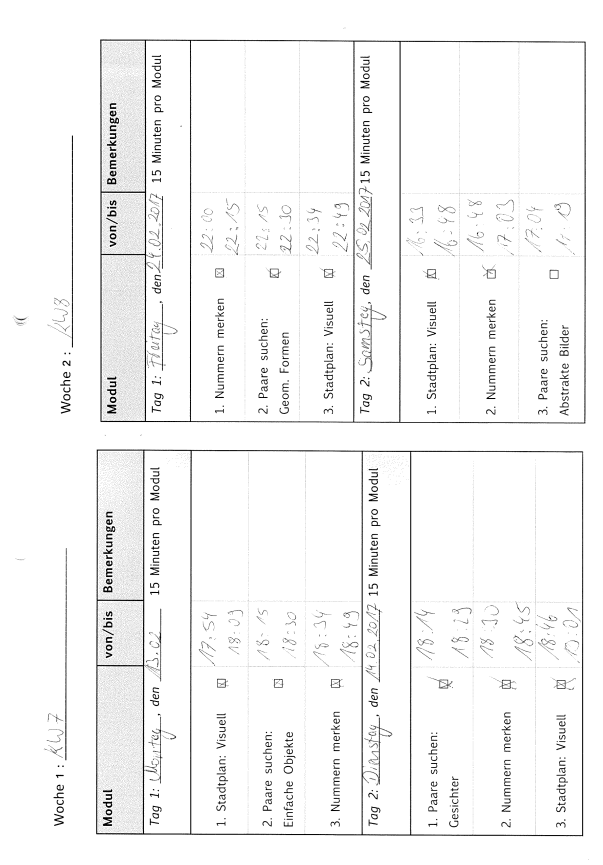


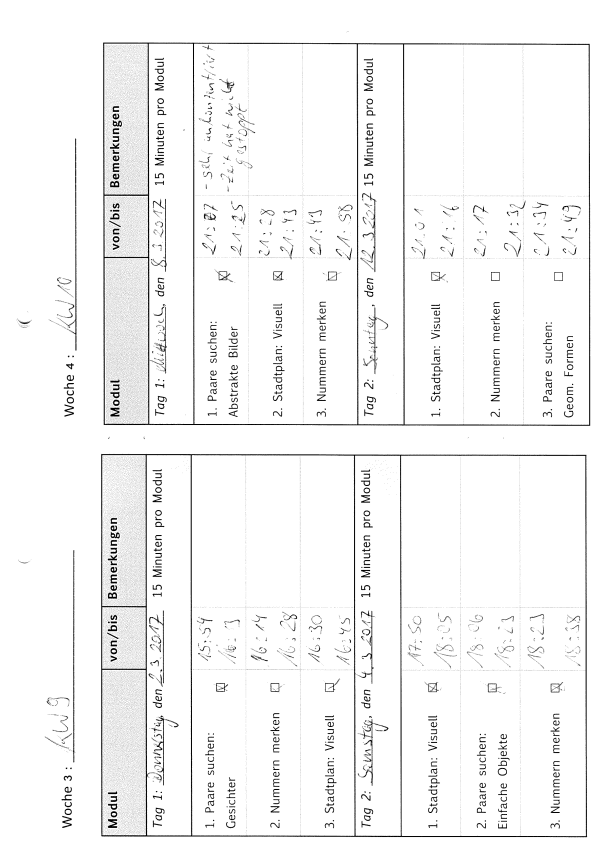


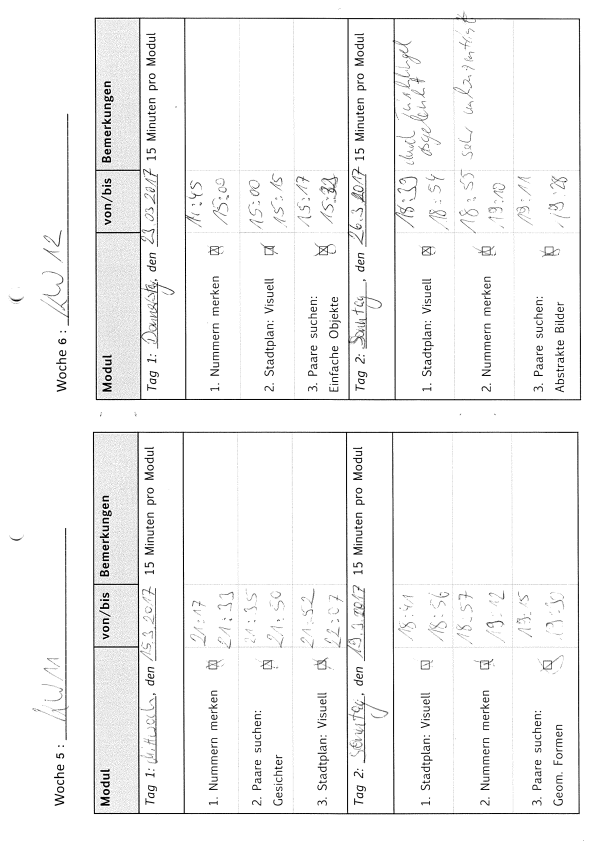


(…) until week 12

Supplement: Supplementary file 3 — Supplementary file3 (DOCX 798 KB) [file 415_2025_13535_MOESM3_ESM.docx]

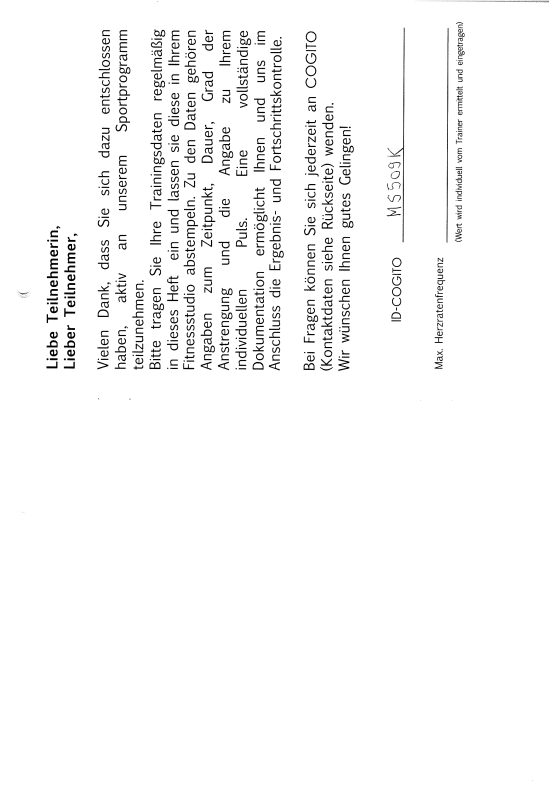
Supplementary File S1. Treadmill Training Log (Example Entry)
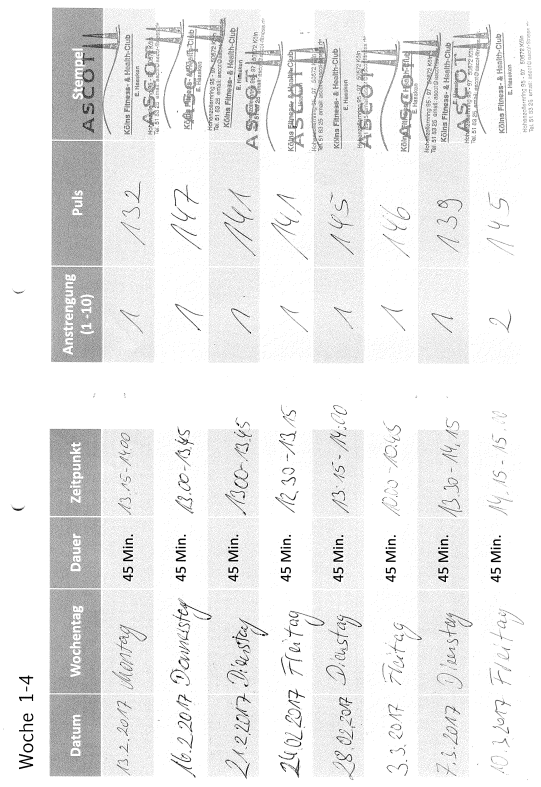

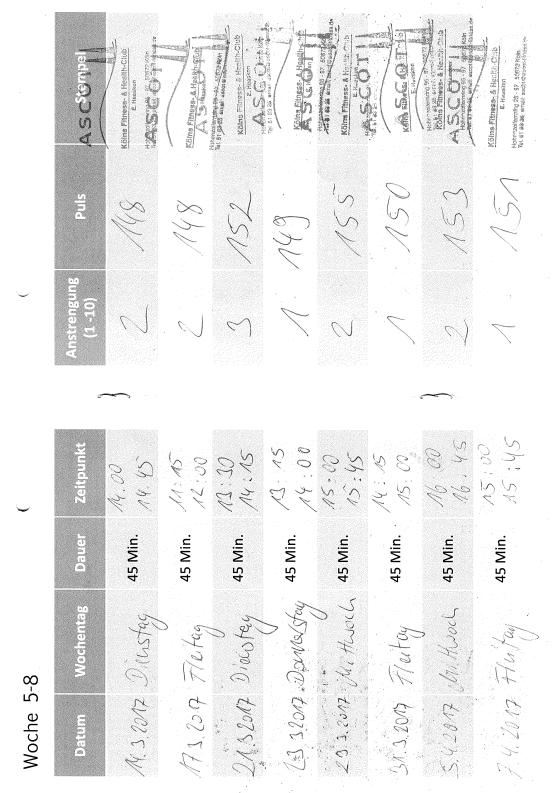


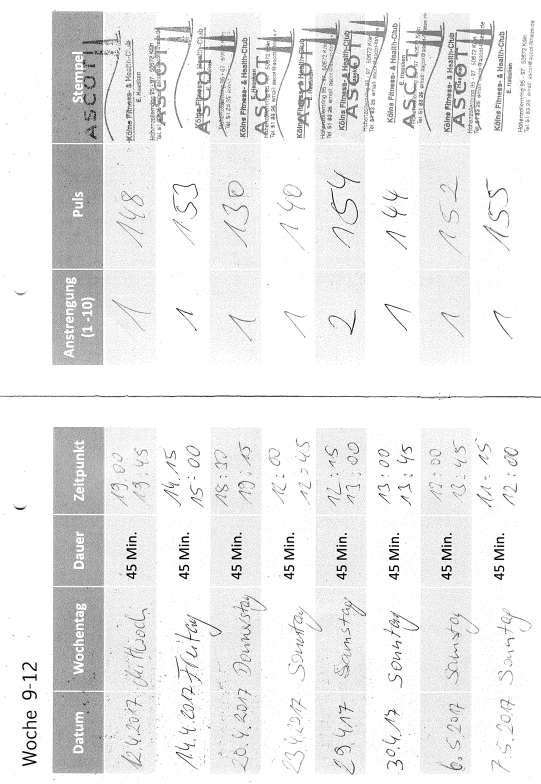

Supplement: Supplementary file 4 — Supplementary file4 (DOCX 1384 KB) [file 415_2025_13535_MOESM4_ESM.docx]
